# A Focus Group Study of Canadian Dairy Farmers' Attitudes and Social Referents on Antimicrobial Use and Antimicrobial Resistance

**DOI:** 10.3389/fvets.2021.645221

**Published:** 2021-06-15

**Authors:** Claudia Cobo-Angel, Stephen J. LeBlanc, Steven M. Roche, Caroline Ritter

**Affiliations:** ^1^Department of Population Medicine, University of Guelph, Guelph, ON, Canada; ^2^Agricultural Communications & Epidemiological Research (ACER) Consulting Limited, Guelph, ON, Canada; ^3^Department of Health Management, University of Prince Edward Island, Charlottetown, PE, Canada

**Keywords:** dairy farm management, dairy producers, dairy cattle, antimicrobial stewardship, qualitative methods

## Abstract

As part of broader actions to combat antimicrobial resistance (AMR), health authorities have promoted the reduction of antimicrobial use (AMU) in food animals. Farmers' attitudes and receptivity to reduction of AMU appear to be variable and context specific. Our research objectives were to gain insight into Canadian dairy farmers' attitudes toward AMU, AMR, and AMU-reduction in the dairy industry, and to explore drivers and barriers to change AMU, including the influence of social referents. We conducted seven focus groups with 42 farmers in two provinces of Canada (New Brunswick and Ontario) and used thematic analysis to identify, analyze, and report patterns in the data. Our results indicate that farmers usually rely on their previous experience and judgement of individual cases of disease when making decisions related to AMU. External referents included other farmers, family members, and veterinarians. However, veterinarians were generally only consulted for unusual cases. Participants in this study expressed that maintaining cattle welfare is their responsibility, and that they were not willing to jeopardize animal welfare in order to reduce AMU. In addition, farmers regarded the cost of investment in improved facilities to prevent disease as an important barrier to reduce AMU. Finally, the majority of participants considered themselves to be low users of antimicrobials and perceived a small role of AMU on dairy farms in AMR. In conclusion, farmers from this study showed self-reliance to decide about AMU on their farms and considered animal-related and economic factors in these decisions. There was a general lack of knowledge of how to reduce AMU without investing in facilities, and there is an opportunity to motivate increased involvement of the veterinarian in AMU-related decisions. These results should be considered to design and refine antimicrobial stewardship programs for dairy farms.

## Introduction

Antimicrobials are important for human and animal health, with indirect effects on food security and food safety. The prevalence of antimicrobial resistance (AMR) in pathogens is a global health challenge for human and veterinary medicine, because infections with resistant pathogens may result in increased severity of disease and infection fatality risk, raising the social, and economic costs of disease (1).

Antimicrobial resistance refers to bacteria acquiring the ability to survive exposure to one or more antimicrobial medicines, resulting in reduction or elimination of the clinical efficacy of the drug against the infection (2). Antimicrobial resistance is an inevitable and natural long-term consequence of exposure to antimicrobials. However, increased selection pressure from overuse or misuse of antimicrobials in humans and animals may accelerate the development of resistant microorganisms (3). Although AMR is an issue that spans human and animal medicine, antimicrobial usage (AMU) in food animal production systems is a known contributing and modifiable factor for the emergence of resistant pathogens of importance to humans (4).

The dairy industry represents one of the largest agri-food industries in Canada, with ~980,000 adult dairy cows on ~10,000 farms (5). Dairy production is spread across the 10 provinces of Canada, with ~70% of the dairy cows in the provinces of Ontario and Quebec. Although the number of dairy farms has been decreasing steadily, total milk production is stable. This is due to an increase of the average herd size (currently 93 milking cows per farm) and modernization of the industry, with increasing proportions of farms with free-stall barn design (currently 35%) and milking robots (currently 12%) (5).

On dairy farms, antimicrobials are used mainly to treat mastitis, reproductive, and respiratory infections (6). Resistant bacteria can be transmitted from cattle to humans through direct contact, as well as through contamination of the environment or food products (6). It is difficult to quantify the routes and risks of transmission of resistant pathogens and genetic elements that confer AMR from animals to humans (4). However, reduction of AMU in food animals has the potential to reduce prevalence of AMR genes in bacteria (7), and appears to have a modest negative effect on AMR in humans; more so for people in direct contact with food animals (1). Therefore, AMU in animals is clearly part of the ecology of AMR in humans.

Consequently, the World Health Organization (WHO), the Food and Agricultural Organization (FAO), and the World Organization for Animal Health (OIE) have promoted strategies to reduce the use of antimicrobials in food animals, especially the drugs classified as critically important for human medicine (8). Some countries have enacted legislation to require reductions in AMU (9). In Canada, similar reductions have not been mandated, but Canadian authorities have strengthened the regulatory framework for veterinary antimicrobial usage, sales, labeling, and importation (10). In addition, antimicrobial stewardship (AMS) was stated as a top priority issue for the Canadian Veterinary Medical Association (11), and reduction of AMU is one of the pillars of AMS (12). The Canadian dairy industry implemented a mandatory national quality assurance program (proAction, https://www.dairyfarmers.ca/proaction), which includes requirements for keeping farm records of medical treatments including AMU (13). Although all antimicrobials are purchased from veterinarians and should be used under veterinary prescription or protocols provided by a veterinarian, dairy farmers have latitude to initiate treatments. There is limited published research on AMU on Canadian dairy farms. Rates of AMU were estimated using garbage can audits in a national study in 2008 (14) and in in the province of Quebec in 2018 (15). Median AMU rate reported by these studies were 316 defined daily doses/100 cow-years (14), and 537 defined courses/100 cow-year (15), respectively. One study in Western Canada investigated producer-reported reasons for AMU in cows, bulls, and calves, indicating that lameness was the most common producer-reported reason for AMU in cows and bulls, and respiratory infections the main reason for AMU in calves (16). Producers' attitudes and referents for AMU in western Canada were explored using a survey in 2013 (17), reporting that farmers' main referent for AMU was their veterinarian. However, Canadian farmers' decision-making process for AMU, their awareness of AMR, and their attitudes and barriers toward reduction of AMU have not been characterized.

The factors that influence dairy farmers' decision-making around AMU are only partially understood. For example, according to the Theory of Planned Behavior (18), a main determinant of peoples' intention to act in a particular way includes attitudes toward that behavior, which are determined by the perceived outcome of the behavior, and the importance of achieving the outcome. Further, intentions and attitudes are influenced by the individual's perceived control over the performance of a behavior (i.e., perceived behavioral control) and assessment of the approval of that behavior from other people whose views are important to them (social referents) (18). However, additional factors such as affect and emotions can further influence decision making (19). Therefore, human decisions are based on a variety of social and psychological constructs such as personality, attitudes, beliefs, values, intentions, skills, knowledge, perceived norms, and perceived self-efficacy, often summarized as mindset (20). Understanding which factors of producers' mindset are influential and modifiable is a first step toward developing effective programs to achieve AMS on dairy farms.

Several methods are available to study the factors influencing AMU among dairy farmers. A recent systematic review (21) including 35 studies of attitudes, knowledge, and awareness of AMU and AMR among dairy farmers and dairy veterinarians reported that more than half of the studies were surveys, and only five studies used focus groups. However, surveys do not allow probing thoughts, views, attitudes, feelings, and experiences of participants (22). Such data may be obtained through qualitative research. Focus groups are group interviews that draw on participants' discussions to generate qualitative data. This method is particularly useful for exploring participants' knowledge, experiences, dominant cultural norms, and shared values (23). Focus groups have been increasingly used in veterinary sciences, particularly One Health, epidemiology, and education (23).

Dairy farmers appear to consider numerous factors in making decisions about AMU. For example, animal health status and animal welfare concerns were important drivers for AMU for interviewed dairy farmers in the U.S. (24, 25). In addition, previous experience and economic factors such as the cost of the disease and milk production have been reported as influential for AMU by interviewed dairy farmers in the UK (26). External drivers of AMU mentioned by dairy farmers in Sweden and the UK include the influence of veterinarians, family, and other farmers (19, 26). Personal factors such as desire for recognition, intrinsic satisfaction of doing a good job, and individual values have been also reported as influential for AMU (27). Clearly, farmers' attitudes and motivations are shaped by social, cultural, economic, and geographic factors such as market prices, regulations, social interactions, and husbandry practices (19). Therefore, we sought to explore the decision-making process around AMU by Canadian dairy farmers. Our objective was to identify some of Canadian dairy farmers' motivations, social referents, and attitudes toward AMU and AMR, and their ideas regarding reduction of AMU in the dairy industry.

## Materials and Methods

### Ethics Statement

This research was approved by the Research Ethics Board of the University of Prince Edward Island (document #6008482). Participants were informed about the study objectives, methods, and implications for them and for the field of study. Informed consent was provided by each participant before starting the sessions. Finally, they were informed about their right to not answer questions that made them feel uncomfortable, to leave the meeting at any time and to contact the research team if they wanted to withdraw their comments from the study. No participants took advantage of these options.

### Positionality and Reflexivity Statement

Positionality refers to the stance or positioning of the researcher in relation to the social and political context of the study. The researcher's positionality impacts the way that data are generated and analyzed (28). All researchers in this study have professional knowledge of dairy farming, three are veterinarians, and all have advanced degrees in epidemiology. None of the authors had prior acquaintance with the participants. The first author (CC-A) led the data analysis and manuscript writing. CC-A is a female, early-career researcher, who has focused her work mainly on infectious diseases and AMR in dairy cattle. As a veterinarian CC-A knows the role of antimicrobials on dairy cattle health and production and understands the complexity of the AMU decisions in dairy farms. Differences in the sociocultural background with participants arise from the fact that CC-A is not Canadian and has never farmed. However, she has experience working with farmers in a variety of settings, environments, and methodologies including in-person surveys and interviews.

### Focus Group Structure and Procedures

Prior to the focus groups, a semi-structured interview guide was developed based on the literature available and field experience of the researchers. The interview guide was reviewed for comprehension by two dairy farmers who did not participate in a focus group, and refined according to their feedback. The interview guide is included in Supplementary Material (Supplementary File 1). The questions and probes sought information on (a) sources of information and social influencers regarding AMU, (b) general considerations when deciding whether to use antimicrobials on their cattle, (c) understanding and opinions about AMR, (d) specific considerations for AMU in calves, and (e) attitudes about reducing AMU in dairy production.

We conducted seven focus groups between February 26, 2020 and March 6, 2020, in two provinces of Canada: four in Ontario, and three in New Brunswick. A convenience sample of participants was recruited. In Ontario, farmers were invited to participate through a personal or e-mail invitation from their local veterinary clinic or their veterinarian. In New Brunswick, 2 individuals involved in the dairy industry as dairy farmers and/or livestock specialists recruited interested participants. In total, 42 dairy farmers participated in the study. There were five to eight participants per focus group. Sessions lasted from 61 to 103 min and were each moderated by one of the researchers (CC-A, CR, or SMR), except for one group (focus group four), which was moderated by an experienced moderator external to the research team. However, a researcher team member (CC-A) co-moderated the session. At the beginning of each focus group, participants completed a short written questionnaire about their farm characteristics. Farmers sat in a circle around a table with the moderators, who introduced the topics to be discussed following the interview guide. The duration and depth of discussion about each topic depended on the farmers. An introductory activity in which farmers and moderators introduced themselves and provided a short description of their work was used as an icebreaker and allowed the transcriptionist to identify and assign quotes to participants. All focus groups were audio recorded and transcribed verbatim by a professional transcriptionist.

#### Data Analysis

One of the authors (CC-A) read the transcripts and simultaneously listened to the audio recordings of the focus groups to verify their quality and accuracy. Thematic analysis was used to identify, analyze, and report patterns within the written data, using a six-phase approach (29). Initial coding was done independently by CC-A and SMR, using a bottom-up, inductive approach aimed at identifying potential themes and subthemes from codes assigned to text elements that informed the research objectives (29). In an iterative process, a codebook (Supplementary File 2) was developed, which was refined through discussion among the authors and adjustment of codes and subthemes until consensus among the authors was reached. Subsequently, two authors (CC-A and SMR) coded the same transcript independently to analyze the clarity and comprehensiveness of the codebook. Intercoder raw agreement percentage was calculated as follows. Briefly, each participant's statement that was assigned to the same code was considered an agreement, and statements assigned to different codes or coded by only one of the coders was considered a disagreement. Raw agreement percentage was calculated dividing the sum of agreements by the sum of agreements and disagreements. Intercoder agreement was 86%. Then, all transcriptions were coded by the same person (CC-A) using NVivo 12 software (QSR International Pty Ltd., 2018).

Unique identifiers (e.g., P2_4) were assigned to quotes from participating farmers with the first number representing the participant id within the focus group (1–8) and the second number representing the session (1–7). Square brackets (i.e., […]) were used to indicate when a quote was shorted or when we inserted explanatory information to ensure the meaning of the quote was maintained. Quotes are reported to illustrate the key features of the subthemes that we identified.

## Results

### Participants and Farm Characteristics

Of the 42 participants, 23 identified as men and 19 as women. The majority of participants were farm owners or co-owners (*n* = 29; 69%), followed by managers (*n* = 10; 24%) or employees of the farm (*n* = 3; 7%). Participants reported a mean (SD) milk production of 34.5 (± 5.7) liters/cow per day and a mean (SD) bulk tank somatic cell count of 135 (± 68) × 10^3^ cells/ml. The demographic characteristics of the farms represented by participants are presented in Table 1.

**Table 1:** Demographic characteristics of the farms represented by participants (*n* = 42) in seven focus groups on antimicrobial use on dairy farms.

**Farm characteristics**	**Number of farms**	**Proportion%**
**Barn design**		
Freestall	36	86
Tiestall	5	12
Mixed	1	2
**Number of full-time employees**		
0	8	19
1	10	24
2–4	20	48
5–8	4	10
**Number of part-time employees**		
0	17	40
1–4	18	43
5–8	6	14
20	1	2
**Number of family members working on farm**		
0	2	5
1–2	19	45
3–4	16	38
5–6	5	12
**Number of lactating cows**		
25–60	8	19
61–120	20	48
121–200	7	17
201–300	4	10
301–480	3	7
**Number of veterinary herd health visits per month**		
<1	6	14
1	7	17
2	24	57
>2	5	12

### Key Themes Identified

**Figure 1:**
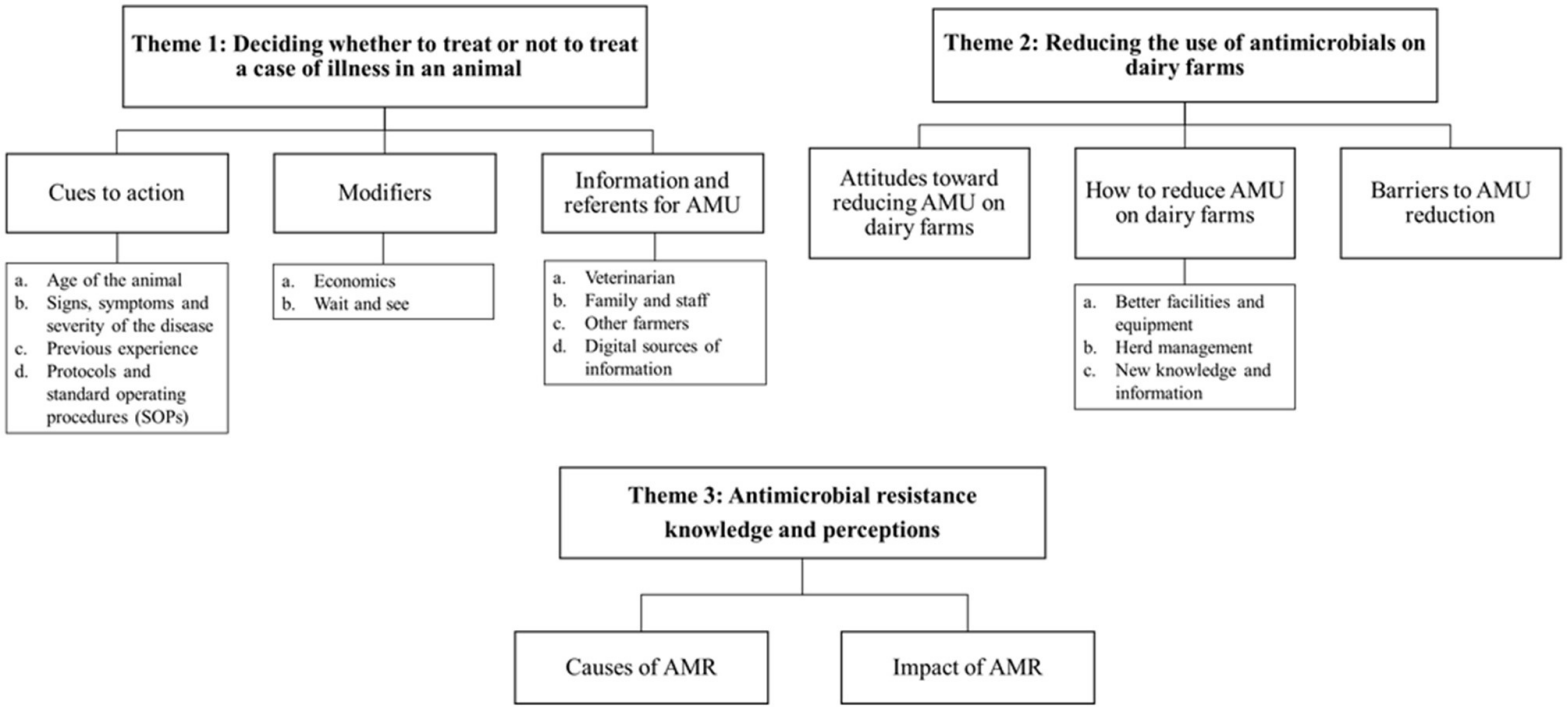
Thematic map of the analysis of data from the seven focus groups of dairy farmers on antimicrobial use in their cattle. AMR, antimicrobial resistance; AMU, antimicrobial usage.

Three key themes were identified from the participants' guided discussions. Theme 1: Deciding whether to treat or not to treat a case of illness in an animal; Theme 2: Reducing the use of antimicrobials on dairy farms; and Theme 3: Antimicrobial resistance knowledge and perceptions. A thematic map of the analysis of data from this study is presented in Figure 1.

#### Theme 1: Deciding Whether to Treat Or Not to Treat a Case of Illness in an Animal

Farmers' decisions about treating their animals with antimicrobials were usually multifactorial. We identified three subthemes from the considerations expressed by the participants (1) Cues to action; (2) Modifiers; and (3) Information and referents for AMU.

#### Subtheme 1: Cues to Action

Farmers usually based their decision on treating an animal with antimicrobials on one or more of the following considerations:

a. Age of the animal

Participants frequently mentioned that they were more likely to treat a calf with antimicrobials than a cow, as expressed in the following quotes*: [P5_3] “For most people and for myself if a calf's sick you treat it, but there's cows that are sick and you make the decision”, [P2_2] “I'd say 99% of the treatments I do is on calves.”* Participants' reasons for having a greater propensity to treat sick calves with antibiotics included more frequent and severe diseases in calves, perception of a stronger immune system or greater resilience in cows, and the lack of a milk withdrawal period after treating calves, as summarized by one of the farmers: [P1_2] “*They're [calves] smaller so they're less able to fight back I think, which is why I would resort to an antibiotic faster in a calf than a cow. I think a calf would get a fever and pneumonia easier than a cow will. Also, I'm not worried about pulling a calf from the milk line, right? I'm not worried about a milk withdrawal.”*

b. Signs, symptoms and severity of the disease

Participants commented that important considerations when deciding about AMU were the signs and symptoms observed in their animals. High temperature was reported by farmers as an alarm sign and therefore it was a trigger for AMU, as reflected by the following statements: [P3_6] “*I think the thermometer is so important. Like if I get a phone call [from an employee] and they say you know this cow is sick, the first question I ask is what's her temperature before we decide how to treat her”;* [P4_1] “*For me temperature is a for sure. If temperature's too high they're going on antibiotics.”* Other signs and symptoms frequently mentioned by farmers as decisive in the AMU decision process included animal behavior, respiratory difficulty, and decreased appetite.

Regarding specific diseases, mastitis in cows, and pneumonia and diarrhea in calves, were frequently stated as the main reasons for AMU on dairy farms. Participants considered some diseases such as pneumonia and retained placenta as severe enough to treat with antimicrobials immediately. Nonetheless, the decision to treat other diseases such as mastitis and diarrhea depended on the severity or the time since the first symptoms appeared, for example: [P7_1] “*I think it depends what the disease is. If it's pneumonia you pretty much need antibiotics, but if it's scours, I would say 80% of the time they don't need antibiotics. But if you see bloody scours, or like a bit of blood in the scours, or if you see prolonged scours then I think it [antimicrobials] would benefit them big time*.” Farmer [P4_7] said, “*Depends what the cow has, right? So RP [retained placenta] gets penicillin protocols, but generally the mastitis [is] like, sample the cow before you treat her.”*

c. Previous experience

Participants expressed that their experience with previous similar cases and the antimicrobials used on those cases played an important role in the AMU decision and the type of antimicrobial used. Their experience also determined the need for external consultation, as indicated by one farmer: [P5_4] “*History is what I would base my decision on - that I've used it in the past and it worked or it didn't work. And if it didn't work maybe I would probably consult the vet to see what he would recommend I use.”*

d. Protocols and standard operating procedures (SOP)

All farmers affirmed having standard operating procedures (SOP) or protocols for AMU, mainly because it is a requirement of the mandatory national milk quality assurance program that is part of proAction. Some farmers relied on those protocols when deciding on antimicrobial treatments for common diseases, as stated by participant [P3_6]: “*On my farm, we have written veterinarian protocols for everything that we do on farm, if a cow's sick and I can figure out what's wrong, I treat her accordingly”* [P1_1]: “*I can say that for calf scours, I would just do my same protocol and I wouldn't consult with anybody*.” However, it was frequently mentioned that they have problems following the SOP. Some of the difficulties discussed by participants included that they only had a general and broad SOP, not useful to apply on specific cases, as expressed by participant [P1_2]: “*So we don't have SOPs as such for specific [cases], like so if there's mastitis there's different protocols we follow depending on the severity….and same with the calves - it's dependent on the animal. There is an SOP written down, but that's probably one of four different methods that I'll use.”* Another problem discussed was that additional or extraneous information makes the protocols difficult to understand, for instance, one farmer [P2_2] explained: “*The other problem with the proAction binder is that there's a lot of extra, unnecessary stuff in there for day-to-day use right? […]. And they [employees] don't need to flip through all of that to find the mastitis treatment protocol.”*

#### Subtheme 2: Modifiers

Despite AMU decisions being generally motivated by one of the reasons described above, other factors might modify the original decision. Modifier factors discussed by participants in this study included:

a. Economics

Farmers might change their decision on treating an animal with antimicrobials based on economic considerations related to the value of the animal, the price of the antimicrobial product, the length of the milk withdrawal period, and the milk production quota situation of the farm at the time, as expressed by [P2_2]: “*I mean you're going to spend 400 bucks on this cow and then you've got to spend drug costs on her and keep the milk out and... So, then it becomes a question of do you want to put all that money into her?.”* Economic factors also affected the decision of the type of antimicrobial used. In particular, milk withdrawal was a concern for participants. For example: [P3_2] “*On a fresh cow if she has an RP [retained placenta], or some pneumonia then you'll first look at Ceftiocyl or Excenel [ceftiofur], with the no milk withdrawal, but if you're over quota you don't need the milk then you'll use a penicillin, which is a cheaper drug. I usually find it's probably cheaper to use penicillin, dump the milk and get the cow healthy quicker, but yeah that definitely makes a difference if I need the milk or not.”*

b. “Wait and see”

Participants discussed that for certain conditions such as mastitis and diarrhea, they preferred to wait from hours to days before treating an animal, expecting a spontaneous clinical improvement, and avoiding antimicrobial treatment. Most of the time, this period was used to give supportive therapy, such as fluids, anti-inflammatory, and/or pain-relief medication. For instance: [P5_2] “*For mastitis, if you can take the pain away and take some of the swelling down, keep them eating and healthy then their immune system will respond.”* [P3_1] “*Same with calves. I'll give them Metacam [an anti-inflammatory medication] […] maybe a good two days and then they still aren't vigorous then yeah I'll start on some sort of an antibiotic protocol, but most of the time if you keep them eating, keep them feeling good it's amazing what they can get over.”*

#### Subtheme 3: Information and Referents for AMU

Participants were asked to discuss the referents and sources of information that influence their decision-making process for treating an animal with antimicrobials. Although participants mentioned various sources, it was commonly indicated that they usually do not consult others before treating common diseases. Instead, farmers mostly use these sources of information when the disease is uncommon, severe, or it did not respond to a first treatment. One farmer commented: [P1_1] “*If it's general things I would not consult anybody, but - because it's the same[…] If it was something new, and I was really kind of wondering what was going on I would maybe go with the vet - but not often*.”

a. Veterinarian

The first response of almost all farmers when they talked about the sources of information and referents regarding AMU was their veterinarian. The main reasons to consult their veterinarian were when they intended to use an antimicrobial off-label, the development of protocols and SOPs, and treatment of uncommon, severe, or recurrent diseases. Farmers also commented that they preferred to text their veterinarian for advice or wait for the scheduled herd health visit to further discuss the cases, rather than ask the veterinarian to visit the farm for a specific disease case. The following comment reflects the relationship between farmers and veterinarian described in the focus groups: [P3_6] “*If the cow's sick I go to the barn, I figure out what's wrong and I treat her accordingly and if I'm not there then I give them [employees] the steps of what to do and then what to treat with. If I am stumped and I can't figure it out, then the vet would be the first one I go to. So, I do herd health every three weeks… So, if I need a prescription, he'll write it, but since proAction has come in I don't do any off-label treatments unless I text him first and get the okay.”* On the other hand, in one of the focus groups, participants expressed frustration regarding the different and sometimes contradictory approaches to treat cattle diseases from different veterinarians. In this regard, one participant said: [P4_3] “*Our vet clinic - I think there's 6 different vets and they have different opinions. You could bring 6 different vets out to look at the same cow and they all give you a different way to treat it.”*

b. Family and staff

Other frequent sources of information discussed by farmers were family and farm staff. These two sources were the same in most cases (i.e., farm staff were family members). In such cases, farmers reported being comfortable consulting, and sharing the AMU decision with them. For example: [P3_1] “*With me it's probably more my husband I kind of bounce stuff off. Like if I'm in the calf barn and I notice something's off I'll get his opinion just to make sure I'm on the right track, but we'll bounce stuff off [each other].”* Conversely, participants whose farm staff were not family members often expressed that employees were sources of information regarding the health status of the animals, but usually they were not involved in the treatment decision, unless they have an established protocol for the specific disease. Many participants commented that this was particularly important when deciding about AMU in cows due to the consequences for milk withdrawal and the risk of bulk tank milk contamination. For example: [P4_2] “*The biggest fear I had when we had employees was that they wouldn't record properly. It wasn't so much that I didn't trust them to administer the right drug, but that if they didn't record it, they didn't put the leg bands on the cow, they didn't make sure the milk was out of the tank, like there's too much stuff. Too much risk and too much room for error.”*

c. Other farmers

Participants considered that other farmers' experience was a valuable source of information regarding AMU in dairy cattle, as expressed in the following quotes: [P1_4] “*I often ask what other producers do... If we have scours [I ask] what are you doing for scours? So yeah a lot of it is working on other guy's farms because that's grassroots results”;* [P5_3] “*That is the nice thing about talking to other producers, you can get ideas on what's being done.”*

d. Digital sources of information

Digital sources of information regarding antimicrobials such as mobile apps, social media, and university and dairy association websites were frequently mentioned by focus group participants. Farmers reported that these sources were used to search for information about antimicrobial compounds, doses, and withdrawal times. Participant [P3_1] commented: “*Compendium of Veterinary Products. It's an app you can get, you can type any kind of disease that you have and it will give you the drugs that you can use for it, or you can type any drug and it will give you all of the uses for it as well as all the meat and milk withdrawals. All the drugs that are licensed for use in Canada are on there.”* In addition, producers reported using social media to interact with other farmers and compare treatment decisions, as expressed by [P5_3]: “*I know there's a couple of groups on Facebook of dairy farmers, and you see sometimes peoples' comments about a treatment and the options people put underneath for treatments... So, I always find interesting to go on the internet seeing what other people use and something I would never use.”*

#### Theme 2: Reducing the Use of Antimicrobials on Dairy Farms

Participants shared their opinions about reducing the use of antimicrobials on dairy farms. From the discussion of this topic, three subthemes were developed: (1) Attitudes toward reducing AMU on dairy farms, (2) Ideas to reduce AMU on dairy farms and (3) Barriers.

#### Subtheme 1: Attitudes Toward Reducing AMU on Dairy Farms

Different attitudes were expressed regarding the idea of reducing the use of antimicrobials on dairy farms. Some participants considered that dairy farmers should be proactively reducing the amount of AMU to meet consumers' and the general public's expectations: [P6_1] “*You're expected to be proactive and try to limit the use of antibiotics by having better facilities. So, I think it would look better on the dairy industry with its supply management if it was proactive and forced farmers to keep up with the current technology available.”* However, other participants stated that they would not change the way they use antimicrobials, unless they were forced by new regulations to reduce AMU, for example: [P4_7] “*The only way that we're going to reduce it [AMU] more in calves is that it's going to get forced on us*.”

Participants in some focus groups mentioned that national and provincial programs directed at improving milk quality have resulted in a progressive reduction of AMU in recent years, e.g., [P6_6] “*I've been at this [dairy farming] a lot of years now and it's really changed a lot … through proAction and CQM [Canadian Quality Milk] before that as much as anything. Like it slowly does change for us, and I think for a lot of producers it changes your mindset. We used to throw Special Formula [an intramammary antimicrobial] at a mastitis case like it was going out of style, like treat them, and treat them, and treat them, and now our vet would say two tubes. Maximum two tubes.”*

Farmers expressed that their responsibilities are not understood by people who are asking for reduced AMU. One participant commented: [P1_1] “*I mean the problem is that they're always saying it's for the consumers and blah, blah, blah. I don't know if they really understand the full extent of what we do. And I don't know if that's really making a huge impact. I mean we do have to do something and be responsible, but at what point are they going to stop?.”*

#### Subtheme 2: How to Reduce AMU on Dairy Farms

Participants had many suggestions to reduce AMU or at least to use antimicrobials prudently on dairy farms. The main categories identified were:

a. Better facilities and equipment

The majority of the suggestions made by participants to reduce the use of antimicrobials were related to the improvement of facilities and the acquisition of new equipment, in order to decrease animal density, as well as improve animal comfort, ventilation, and cleanliness. For example, participants said: [P4_1] “*The newer barns are better equipped, better ventilation which will prevent - or sorry which will mean it'll be hopefully less antibiotics having to be put out.”* [P7_1] “*I think that an easier, more efficient way of reducing antibiotics is improving quality of the facilities and like for example we put in air bags back in December and on the calf study we went from… between 30% and 40% calves treated with pneumonia. We went from that to 8% in like November, December.”*

In addition, farmers commented that robots and automated milking systems are useful to keep a healthier herd, since it is possible to detect abnormalities earlier and take corrective action. One farmer commented: [P3_6] “*We have a relatively new tool to us and that's because of the robot. We've got a lot of data that we didn't have before […]and every cow that gets milked you get a new set of data. So, I found in the last year we've changed our approach to the antibiotics a fair bit because we have that data. There's a health report and the first thing I do every morning before I make my cup of tea is I check the health report and see what might have happened the night before […] And I don't have statistics, but just anecdotally we've used an incredibly smaller amount of mastitis drugs in the last year than we used previously*.” The key indicators that participants reported as being helpful to detect diseases in the early stages were reduced activity or feed consumption, increased temperature, increase in milk somatic cell counts, and changes in milk conductivity.

b. Herd management

Farmers discussed several options to keep cattle healthy through herd management, such as improving bedding, ventilation, and cleanliness of facilities and equipment. An additional common preventive practice mentioned was vaccination. One participant expressed: [P2_2] “*How much antibiotic use could be prevented if everybody did have a vaccination protocol in place?.”* Calf-specific suggestions included feeding more milk, and keeping clean and dry calf pens, as advocated by one of the participants: [P6_1] “*In my opinion, this is my experience, feed more milk. If you give more milk it doesn't cost that much more, and you save so much effort after the fact.”*

Selection of bulls or semen based on specific health traits was also suggested as a way of reducing AMU. Some farmers reported the use of calving-ease bulls to reduce dystocia, particularly in heifers, as expressed by one participant [P5_3] “*We've only used calving-ease bulls on heifers for a long time and that just streamlines the calving as far as heifers go.”* Other farmers commented that using semen from a specific program with high immune trait reduced the incidence of certain diseases and therefore AMU: [P1_1] “*If you use Immunity Plus bulls that we've been using for years it was 30% less scours.”*

c. New knowledge and information

Participants expressed that new information and training regarding herd management, AMS and AMR were important complements to improved facilities and improved herd management to reduce AMU in dairy farms. One participant said: [P5_3] “*you still have to go to the point of making sure there's prudent use of it [antimicrobials] and dosages are correct and you're not treating conditions that aren't actually receptive to antibiotics […] that's where I think education is needed.”* Other participants suggested a periodic review of the protocols and antimicrobial recommendations would be beneficial to reduce AMU, for instance: [P6_1] “*I think you buy your drugs from the vet. So, I think when the vet sells you the drugs say hey, what are you using this for? Oh, for the scours? And then maybe the vet could say, oh, let's take a look. And maybe the DFO [Dairy Farmers of Ontario] could give each farmer a half an hour consultation a year with the vet to try to reduce their antibiotic use.”*

Among the ideas to reduce AMU on farms, focus group participants mentioned different ways to compare the AMU on their farm with other farms in order to know if they are high users of antimicrobials. For example: [P3_3] “*I don't really know how I compare to other people. I mean I know how my two-year old's calving so that's kind of a good indication but if you knew, okay, this is the level of antibiotic use you are per kilo I'm way too high, right?”* Another producer suggested a similar idea but comparing the amount of money they spend on antimicrobials, as follows: [P5_2] “*They [Dairy Farmers of Ontario] do analysis every year as part of what they use for the cost of production formula for the pricing of milk…So if you look up that then you can also compare your dairy farm books to this data set of farms… if there was a separate category for - right now it just says vets and medicine. There was a separate category for antibiotics, right? Then you could be like whoa, we're way above. Oh, we're doing pretty good like just being able to separate that out as a bill mentally brings awareness to it and makes it something that you can act on. Right now, it's kind of buried in the data and it's not something that comes to the forefront.”*

#### Subtheme 3: Barriers

Farmers discussed that most of the opportunities they perceived to reduce AMU on dairy farms required substantial economic investment and that was recognized as the main barrier to reduce AMU. One farmer commented: [P2_3] “*Maybe some people can't quite, you know, afford the big step or initial investment to do something but then… if your cash flow is tight and you want to spend say 30 grand yeah. So, you'd rather treat 10 calves.”* Another participant stated: [P2_5] “*I think for us the economics of being able to - having a housing system that's easier to clean or to have less calves in areas and stuff it - we just don't have the money to do those things.”*

Most participants considered themselves low users of antimicrobials, and felt it was not feasible to further reduce AMU without affecting animal health and welfare, as expressed in the following: [P3_2] “*I do think like with antibiotic usage from an animal welfare standpoint it's not justifiable not to treat the cows. If we can help them, and we can fix them you know we can get them through this with antibiotics I think we should”;* [P5_3] “*Well if the calf needs antibiotics and they're telling me to use less then I guess that's a problem welfare wise. Like the calf is sick and they're telling me not to treat her?.”*

In addition, farmers frequently expressed that information and education given through meetings and conferences was repetitive. [P2_5] explained: “*Our vet clinic got like this dairy day that they do every March or something like that and honestly we've kind of stopped going to it because it's a lot of the same information year after year and I'm like, yeah dudes, like we know this. But then when I talk to other guys in the room who are like: you feed your calves more than 6 litres of milk a day? And I'm like: jeez, how are we still talking about this? Like we're talking about this year after year and people still just aren't doing it. So, like the knowledge is there but somehow the uptake is real slow.”* As reflected in the previous quote and other comments, participants felt that some farmers are not interested in changing their practices and their way of using antimicrobials, therefore they do not attend conferences or look for new information, as expressed in the following comment: [P1_2] “*Whereas you guys might look at your antibiotic use and be like: yeah, we keep it as low as we can, and we only use the applicable stuff well that's great… but I'm pretty sure there's a large percentage of the population that doesn't share that mindset but you never meet them because they never come to things like this. And they never go to the Healthy Calf conference [a regional extension meeting].”* Another barrier expressed by participants was the technical language used in the information regarding antimicrobials. One participant found language used by professionals during conferences and meetings confusing: [P1_7] “*What I find quite often is when you go to places where there's people like you guys who've done all the university, you know they talk about things and then I think what did they say? What is that? You know? It's a language thing.”*

It was stated by some farmers that the Canadian supply management system, characterized by matching the national supply of milk to demand, with farm-specific quotas (daily kg of butterfat that each farmer is to produce), was a barrier to improve management practices and therefore to reduce AMU, because the system offers a degree of economic stability, which according to participants does not encourage the adoption of new and better farming practices. Some of the comments in this regard were: [P2_2*] “I appreciate the system for the stability that it provides but quota breeds lazy farmers. As much as I love the quota system, as long as it's around those people [lazy farmers] will be around because they can economically make it”;* Another producer commented: [P2_7] “*Without that quota system you'd be forced to get a lot more efficient whether you wanted to or not.”* However, other farmers argued that the barrier for improving farming practices was the attitude of some producers and not the supply management system.

#### Theme 3. Antimicrobial Resistance Knowledge and Perceptions

When farmers were asked about their thoughts about AMR, most of them were familiar with the concept. One farmer explained: [P3_1] “*The bugs have become like immune to that drug that when you do treat them with that antibiotic they're not responding in the same way they once had or are used to.”* Two subthemes were identified from their discussion at this regard: causes of AMR and impact of AMR.

#### Subtheme 1: Causes of AMR

The first cause of AMR that usually came out in the discussion was overuse of antibiotics in dairy production. For instance, one farmer said: [P3_5] “*If you're overusing that drug then there's going to be resistance to it and it's not going to work, it's not going to cure*.” However, when discussion continued, farmers mentioned other causes such as incomplete treatment and the use of the same antimicrobial compound for long periods of time.

Farmers expressed not being concerned about feeding calves with waste milk with antimicrobial residues as a cause of AMR. They considered that the main problem would be direct treatment of animals with antimicrobials, as indicated in the following statement: [P5_3] “*I think the bigger risk is treating calves with antibiotics, or cows with antibiotics and getting it directly into the meat. I'd be more concerned with how you're treating cows and resistance in the food population than I would be in feeding waste milk to calves.”* Conversely, the ingestion of antimicrobials through medicated feed was considered an important cause of AMR by participants. One farmer said: “*if you put it [antimicrobials] in your feed… small doses it does keep the infection down but it also - I imagine they would build up - they would get used to that little bit and yeah the bacteria would just get a little bit stronger and overcome.”*

Although our interview guide did not include the topic of AMR in humans, all focus groups discussed the role of antimicrobials in human medicine on AMR. Participants mentioned that AMU in human medicine was an issue especially in Canada: [P8_5] “*It's a problem and this is a whole different discussion and you know is it a problem coming from where? That's the problem. That's the discussion. Because I know like in Europe when a baby has an ear infection and you go to the hospital, go home. And here, in Canada they get penicillin every time they have an ear infection.”* Another farmer commented: [P9_5] “*Quebec actually is known for over-prescribing all the time with babies and that's for all the MRSA [methicillin-resistant Staphylococcus aureus] as well.”*

It was also mentioned that agriculture was receiving all the attention regarding AMR, instead of human medicine. For example: [P5_2] “*The whole conversation of resistance is agriculture we need to do something yes, but human medicine has to clean up their system first right?*”; [P5_3] “*I keep hearing in the media talking about antibiotic usage on farms and yet they dismiss any talk of anything supportive as bunk when it comes to people. And you're a bad farmer if you use antibiotics on your cattle, but you'll get your kids taken away if you don't use antibiotics on your sick kid.”*

#### Subtheme 2. Impact of AMR

Participants considered that currently AMR is not a big problem in dairy production, although it was commonly mentioned that mastitis was a difficult disease to treat. Farmers commented that AMR was more problematic for human health than for animal health: [P4_4] “*I don't know if it's as important with animals as much as people because you're trying to eliminate your antibiotics in your food, your food chain, so it doesn't show up - like the residues don't show up in humans.”* However, some participants expressed concern about the impact of farming on the AMR for humans, as expressed in the following quotes: [P5_6] “*I'm more concerned about resistance to drugs that are drugs that are also used in humans. So, if it's you know a penicillin for instance it's a very common drug used in humans I'm more concerned about over-using that in my cow herd if I may have an impact on, you know, some super bug 10 years down the road or 20 years down the road that, you know, it's in our hospitals and people are dying because we've contributed to this problem on our farms.”*

[P5_1] “*It's a bigger picture thing. It's not about maybe doing a better job with your animals. It's about whether they're going to be effective for everybody.”* Other farmers affirmed that they try to avoid certain antimicrobials due to their importance for human health: [P2_5] “*It's very important for humans so we don't really use Excenel [ceftiofur].”*

## Discussion

We explored the attitudes, external referents, and sources of information that influence Canadian dairy farmers in their decisions regarding AMU and their views about AMR. Our main findings were: (1) Farmers' personal perceived knowledge and experience diagnosing and treating common diseases are the main factors considered when deciding about AMU, and the role of veterinarian as an external referent for AMU is limited almost exclusively to treat uncommon or chronic cases; (2) Farmers lack knowledge of how to reduce AMU without investing in facilities; and (3) Awareness of the impact of AMU on AMR is low among dairy farmers.

Farmers in this study showed considerable self-reliance to diagnose and treat their animals with antimicrobials. They usually rely on their previous experience and judgement of the particular case based on signs, symptoms, age of the animal, and perceived severity of the diseases to decide the AMU or the need for external consultation. The ability to take good care of their animals is important to farmers because it is associated with the concept of being a “good farmer” (i.e., being accepted and appreciated by their peers) (30). This is consistent with previous research in other contexts (19, 24, 25). Conforming to the perceived social norms of other dairy farmers and secondarily of their veterinarian was influential in antimicrobial treatment decisions for Dutch dairy farmers (31).

Studies from Europe and North America have reported that veterinary advice is the most influential factor for AMU decision by dairy farmers (25, 32). However, in this study, many participants reported consulting with the veterinarian only in cases of recurrent, severe, or uncommon disease or after trying different therapeutic options, indicating that the role of the veterinarian in deciding whether to administer antimicrobial treatments to cattle may be infrequent and consequently less influential. In fact, dairy farmers frequently described consulting family members and other farmers before consulting the veterinarian. A previous quantitative study of AMU in calves in Western Canada, reported that dairy farmers preferred switching products before calling the veterinarian when antimicrobial treatment failed (17). These results indicate that there is room for improvement in the veterinarian-farmer relationship in Canadian dairy industry, and that improvement of the relationship could improve AMS. Although improving AMS is considered a top priority for both veterinarians and dairy farmers in Canada (33), the lack of communication and between farmers and veterinarians is a barrier to that goal (34). In this study, some farmers considered technical language and varying opinions and advices on AMU from veterinarians and other advisors as barriers for prudent AMU. It is known that communication between farmers and veterinarians is often suboptimal (20). Therefore, it is necessary to work on the veterinarians' communication strategies for knowledge translation and transfer. Community engagement (i.e., involving and collaborating with the community to address issues that affect the community) has been a cornerstone of human health interventions to reduce smoking, obesity, and heart disease (35). Similar outcomes could be expected in veterinary medicine, in particular related to reduction of AMU. Involving farmers in the design and development of courses, meetings, and other knowledge transfer strategies could improve their level of engagement and the relevance of the content offered. This might motivate them to improve AMU practices, because it builds trust between farmers and advisors, enlists new resources and allies, and improves communication. Successful community engagement projects evolve into lasting collaborations (35).

New technologies such as milking robots and wearable sensors, and digital sources of information such as social media, websites, and mobile apps are influential in farmers' decision-making process. Although the new communications technologies could offer a channel for valid information regarding animal health and AMU, the risk of misinformation on social media and other informal websites is high (32). Therefore, veterinarians and other advisors need to promote the use of evidence-based information among farmers, including online sources to minimize misinformation risks. There is a need for additional research on how to apply data from precision technologies to AMU decisions, and an opportunity for veterinarians to become better informed and involved with application of these data by farmers.

According to our results, calves are more likely to be treated with antimicrobials than cows. This result is consistent with a recent study in Western Canada, where dairy farmers reported the use of more antimicrobial doses for disease prevention and treatment in young stock than in adult cows (16). Farmers' reasons for more frequent AMU in calves included considerations of the calves' immature immune system and lack of concern about milk withdrawal. When treating cows, participants often preferred to use ceftiofur-based products, which is the only antimicrobial with a zero-day milk withdrawal time, in order to avoid milk withholding periods. Third and higher generation cephalosporins, such as ceftiofur are considered critically important antimicrobials (CIAs) in human medicine (36), yet ceftiofur has been reported as one of the most commonly used antimicrobials in dairy cows around the world, presumably due to the zero-day withdrawal period for milk (37). High priority CIA represented a median of 17% of AMU on dairy farms in Quebec (23). Cephalosporins and penicillins were among the five most commonly used antimicrobial classes on dairy farms in Canada (14). These studies were conducted before Canada's new framework for action on antimicrobial resistance and antimicrobial use. The framework includes a strengthened regulation for using medically important antimicrobials in agriculture. The new regulations includes mandatory veterinary prescription for medically important antimicrobials, prohibition of their use for growth promotion, and mandatory sales reporting of these antimicrobials (10). New studies of AMU on dairy farms in Canada are needed to evaluate the effect of the new regulations.

Even though economic considerations influenced AMU decisions, particularly when treating cows, participants from this study also placed a high value on animal welfare. They expressed that maintaining cattle welfare is their responsibility, and that they are not willing to jeopardize animal welfare in order to reduce AMU. Previous research suggests that farmers have emotional reasons to treat cattle with antimicrobials, such as personal attachment, and a moral obligation of preventing animals from suffering (19, 24). Further, farmers are willing to compromise economic profit to some extent in order to improve animal health and welfare (19, 38). The impact of reducing AMU on animal welfare is difficult to determine, and research focuses generally on the relationship between AMU and animal health. In the Netherlands, a combination of compulsory and voluntary actions to reduce AMU in dairy cattle resulted in a 56% reduction in AMU without major impacts on animal health and productivity (39). However, a recent systematic review on the unintended consequences of AMU reduction in food producing animals reported that effects on animal health were inconsistent among studies (40).

There are two major routes to reduce AMU on dairy farms. The first is to minimize the incidence of infectious disease at the farm level through optimal housing and nutrition, good hygiene and biosecurity, and vaccination. The second route is prudent AMU when treatment is indicated based on proper diagnosis and understanding the efficacy and the pharmacology of the antimicrobials used (39). The vast majority of our participants tended to focus on the first route. Most of their suggestions were related to the improvement of facilities, the acquisition of new equipment, and better herd management to keep animals healthy. Fewer farmers suggested revision of AMU protocols and none of them mentioned microbiological culture or antimicrobial susceptibility testing. This could be related to the fact that generally farmers diagnose their animals based on clinical signs. There is an opportunity to use antimicrobials more prudently by improving farmers' training in basic diagnosis and treatment.

Other suggestions for motivating a reduction in AMU included comparisons of AMU between farms (i.e., “benchmarking”). This could be related to the concept of the “good farmer” whereby farmers strive for cultural capital (i.e., to be viewed by their farmer peers as a leader or someone who does the right thing) (27). Interventions to motivate AMU reductions by appealing to farmers' intrinsic values and motivations seem more likely to succeed rather than appeals based on broader social perceptions or forced behavioral changes (39). In particular, benchmarking has previously been demonstrated to improve calf management on Canadian dairy farms (34). Enforcement of rules and regulations as the sole intervention had a limited effect to reduce AMU in human medicine (41). In the Netherlands, AMU on dairy farms was reduced by 70% through legislation, but this accomplishment was attributed to an approach that included appeals to a variety of motivations that were relevant to dairy farmers (9). According to The Model of Action Phases, pursuit of a goal (e.g., reducing AMU) is characterized by motivational and volitional phases. The motivational phase is pre-decisional and is characterized by deliberation (i.e., farmers have not yet decided to reduce AMU and they have to weigh pros and cons) (42). The volitional phase translates motivation into actions to achieve the goal (e.g., creating protocols or improving biosecurity to reduce AMU), and the evaluation of the effectiveness of those measures to accomplish the goals. It is important to understand which phase farmers are in when considering how to motivate behavioral change, because each phase needs a different kind of support. In this study, most farmers seemed to be in the pre-decisional phase. In this phase, individuals need to consider information on the feasibility (the expectation that the goal will succeed) and the desirability (the value of the expected outcome) of making a change (43). Therefore, providing clear and accurate information about the practicality and potential benefits of reducing AMU would likely be influential in motivating farmers to go from deliberation to implementation of plans to reduce AMU.

Farmers perceived that the cost of improving facilities and equipment (which they perceived to the key factors in reducing the need for AMU) was the main barrier to reduce AMU. Similar limitations have been described in studies from the United Kingdom and Sweden (19, 26). Although reducing AMU could decrease the drug costs of dairy farms, production costs could potentially be higher. One study estimated that the average increase in costs in the scenarios of prohibition and reduced AMU would be US$150 per cow per year and US$61 per cow per year, respectively (44). Extra costs are associated principally with higher prevalence of diseases, extended days open, cow replacements, and reduced milk production (44). Therefore, cost-effective strategies are necessary to motivate farmers to reduce AMU. Importantly, there are strategies that could reduce AMU on Canadian dairy farms without large economic investments on facilities, such as increasing veterinary involvement in AMU decisions. Although veterinary consultation for each disease case on the farm is not practical, producers and their veterinarians can work together to design, and periodically update tailored protocols for diagnosing and treating common diseases on dairy farms such as mastitis, metritis, and calf diarrhea. Similarly, revision and updating biosecurity and hygiene measures would be expected to reduce the incidence of infections and consequently the use of antimicrobials. Interventions aimed at improving udder health, uterine and replacement calf health successfully achieved reduced AMU on dairy farms in Switzerland (45).

The majority of participants in this study were familiar with the concept of AMR and some of its causes. However, AMR in dairy cattle was not seen to be a current problem. Participants were more aware of the impacts of AMR on human health than its impacts on animal health. However, most of them were skeptical about the magnitude of the role of AMU in agriculture and specifically of dairy farming on AMR in human pathogens. Prior research indicates that farmers are less willing to modify their management practices if they do not see or accept the negative implications of their current behavior (46). Consistent with previous studies in different settings, most farmers considered themselves low users of antimicrobials (24, 47), indicating that they do not perceive themselves as part of the AMR problem. It would be useful to understand the magnitude of the gap between farmers' perceived and actual AMU by comparing this estimate with AMU data in future studies. The difficulty of quantifying the effect of AMU in veterinary medicine and specifically in dairy farming on AMR in humans makes it difficult to argue this question from evidence. Nonetheless, it seems clear that social, market, and regulatory pressures to reduce AMU will only increase. In any case, some farmers affirmed that they would avoid certain antimicrobials if they knew they are important for human medicine, which represents an opportunity to motivate farmers to be more selective when choosing antimicrobials by offering them information about the classification of antimicrobials and the consequences of AMR to high priority antimicrobials.

Qualitative research seeks to address the nature of the phenomena rather than measure them (22). Therefore, qualitative research does not intend to provide a representative sample of the target population and does not lead to the same kinds of inferences as quantitative methods (22). We employed focus groups to explore the reasons behind AMU and to bring out the complexity and diversity of opinions and thinking on AMU and AMR. However, sampling for focus groups may be susceptible to self-selection bias: our participants might be more informed or hold stronger opinions about AMU or AMR than non-participants. We note that a greater proportion of participants were from farms with free-stall barns than in the source population of Ontario and New Brunswick where 33 and 51% of the of farms have free-stall barns (5). Therefore, their views may possibly be more reflective of modern dairy farms. It would be interesting to explore the views, values, attitudes and motivations of farmers who are high users of antimicrobials to explore additional measures that could be taken in order to reduce their AMU.

In addition, we acknowledge that social referents are technically any individual, group, or entity that might influence an individual's mindset regarding a particular issue or practice (e.g., consumers or public opinion) (18). However, we asked participants specifically for their direct referents advisors who influenced their decisions regarding animal health and AMU. We did not exclude comments about how consumers or other stakeholders in the value chain might influence them, but we did not probe and explore all potential social referents. It would be interesting to explore Canadian farmers' indirect social referents in future studies. Finally, the participants in the focus groups in Ontario had taken part in a focus group on calf management shortly before this study (separated by a lunch break), which could have affected their responses in our discussions. However, we did not observe differences in the themes identified between groups in Ontario and New Brunswick or between groups moderated by researchers and the external moderator.

## Conclusion

In conclusion, attitudes toward AMU and AMR among Canadian dairy farmers involve multiple influences including social, economical, and animal-related factors. Farmers' own observations and experiences were central to their AMU decisions, with referents from peers and family, and lesser influence from veterinarians and written protocols. The main barriers to reduced AMU were concern for animal welfare and the economic difficulty of infrastructure changes that were perceived to be necessary to reduce the burden of disease. Programs aimed at improving AMS should be designed considering these factors, in particular the apparent importance of farmers' empirical experience in their decision-making. Because most farmers considered themselves to be low and responsible users of antimicrobials, benchmarking data on AMU may be one useful element of a program to change behavior. Future research should refine and quantify the relative importance of self-reliance and external norms and referents in dairy farmers' AMU behaviors.

## Data Availability Statement

The raw data cannot be shared publicly because it contains sensitive information from qualitative interviews. Full interview transcripts cannot be shared due to the terms of the participants' consent. Some transcript excerpts are included in the text of the article.

## Ethics Statement

The study was reviewed and approved by the Research Ethics Board of the University of Prince Edward Island (document #6008482). The patients/participants provided their written informed consent to participate in this study.

## Author Contributions

CC-A contributed to data collection, analysis, and writing of the first draft of the manuscript. The interview guide was designed by CR and reviewed and edited by all the authors. Focus groups were conducted by CC-A, CR, and SR. All authors contributed to data analysis, manuscript writing and revision, and approved the submitted version.

## Conflict of Interest

SR owns ACER Consulting Ltd. The remaining authors declare that the research was conducted in the absence of any commercial or financial relationships that could be construed as a potential conflict of interest. The editor declared a past co-authorship with one of the reviewer IM and confirmed the absence of any ongoing collaboration during review.
