# Supplementary material for: A Focus Group Study of Canadian Dairy Farmers' Attitudes and Social Referents on Antimicrobial Use and Antimicrobial Resistance
Source: Front Vet Sci. 2021 Jun 15;8:645221. doi: 10.3389/fvets.2021.645221 (PMC8239135; doi:10.3389/fvets.2021.645221)
Supplement: Supplementary file 1 [file Data_Sheet_1.PDF]

**A focus group study of Canadian dairy farmers' attitudes and referents on antimicrobial usage and antimicrobial resistance**

# Focus Groups – Semi-structured discussion guide

**PART 1) Social Network (20-30min)**

<u>*Introduction first part:*</u>
*We are here today to talk about use of antibiotics. However, first, I would like to know more about your health management in general and who you consult with. That is important to see the information you provide later in the larger context.*

1) Post-it exercise – Who do you talk to about animal health on your farm, for example, to discuss a certain issue or before you make a decision regarding animal health? Please use the sticky notes and place more important (influential) people closer to the middle. The idea is that as a group you can agree on certain people and discuss where they should end up on the bull's eye. But if you have someone you regularly talk to and that is not shared with the group, that is great, too. I recognize that this is not the same for everyone, but I would like to use this activity to stimulate a bit of a discussion.

2) While participants are placing the social referents, facilitators encourage conversation, *for example:*

- *Why did you place xyz there? Is that the same for everyone?*
- *What role do family members play compared to professional farm advisors?*
- *How do you decide who you talk to about your animal health?*
- *In what situations do you talk to your employee about the health of your herd?*

3) To what extent do the people you listed here consult with each other, either with or without you present, about your farm? Please draw lines between the people that talk to each other about animal health on your farm.

- Where are the strongest connections?
- How often do the people you listed here consult with each other?
- Are there regular meetings scheduled to discuss your farm?
- How do you feel about the level of communication?

**TAKE PICTURE!!!**

4) How would the people in the bull's eye change when we replace general "Animal Health" with decisions about antibiotic use? Who stays, who goes, and who would you add?

- Who are the main decision-makers when it comes to antibiotic treatments on your farm?
    - Under which circumstances do you consult other people before using antibiotics?
    - What is the influence of others on your decision?
    - Do your employees use antibiotics independently?

**A focus group study of Canadian dairy farmers' attitudes and referents on antimicrobial usage and antimicrobial resistance**

**PART 2 – Antibiotic Use**

a) GENERAL (20-30min) – *I would now like to learn a bit more about how you make decisions around antibiotic use in general, without being too specific about certain diseases or applications*

- In general, how do you decide whether or not to use an antibiotic? What considerations go into your decision-making process?
    - Do you have written SOP's for antibiotic treatments?
        - When do use them? When or why not?

- How do you decide which antibiotic you use?
    - Does whether you are under or over quota play a role in your decision?
    - How does the fact that you have some antibiotics on farm influence your decision to use a particular antibiotic?

- How do you decide on the duration and dose of the antibiotic use? (Probe to cover both dose and duration)
    - How do you feel about the information provided on the labels of the antibiotics?
        - Do you follow the recommendations on the labels? Why (not)?

- How confident do you feel that you are using antibiotics correctly?
    - In which areas do you feel most / least confident?

- Can you tell me what you understand by "antibiotic resistance"?
    - What do you think causes antibiotic resistance?
        - To what extent do you think resistance to antibiotics is a problem?
        - Why or why not should the dairy industry try to reduce use of antibiotics?

b) CALVES *(>=30min) - As part of our research we are especially interested in the use of antibiotics in calves*

- What do you think are the most common routes for calves to be exposed to antibiotics?
    - What do you mostly use antibiotics for in calves?
        - Do you use antibiotics in calves preventatively or for treatment purposes?
        - To what extent do you think ingestion of antibiotics through milk or milk replacer plays a role?

- How do you decide whether or not to use an antibiotic for a calf or calves?
    - What do you base your decision on whether you administer antibiotics to a calf or not?
        - Remember the last time you administered an antibiotic to a calf or calves how did you arrive at that decision?

  - When there is a calf that appears sick, how long do you wait before using antibiotics?

- Is your decision process on the type of the antibiotic, duration and dosage similar or different from what we discussed previously? (Refer back to what was discussed)
  - Do you have standard operating procedures (SOPs) or protocols for the use of antibiotics in calves?
  - What do you do if a calf does not seem to respond to antibiotic treatment?

- What do you think might be advantages of reducing antibiotic use in calves?
- What do you think might be disadvantages of reducing antibiotic use in calves?

- To what extent do you think it is possible to be more prudent regarding the use of antibiotics in calves?
  - What are specific applications where you think it is most possible to be more prudent regarding the use of antibiotics in calves?
  - Have you tried it before and if so, what was your experience?
  - What could be alternatives to the use of antibiotics in calves?
    - Do you think these alternatives are feasible?

- What (if anything) would have to be changed to be more prudent in the use of antibiotics in calves?
  - What kind of support/information would you need to make this happen?
