# Supplementary material for: A Focus Group Study of Canadian Dairy Farmers' Attitudes and Social Referents on Antimicrobial Use and Antimicrobial Resistance
Source: Front Vet Sci. 2021 Jun 15;8:645221. doi: 10.3389/fvets.2021.645221 (PMC8239135; doi:10.3389/fvets.2021.645221)
Supplement: Supplementary file 2 [file Data_Sheet_2.PDF]

A focus group study of Canadian dairy farmers' attitudes and referents on antimicrobial usage and antimicrobial resistance

Codebook used to analyze qualitative data from seven focus groups

Name	Description
**Theme 1: Deciding whether to treat or not to treat a case of illness in an animal**	This theme refers to the drivers behind the decision of treating animals with antimicrobials. Specific considerations and common situations described by the farmers when deciding the use of antimicrobials in their farms
Subtheme 1: Cues to action	Factors that motivate the antimicrobials use in the dairy farms
a. Age of the animal	Participants considerations related to the age of the animal when deciding whether to use or not antimicrobials in their farms
b. Signs, symptoms, and diseases	Participants' considerations in the antimicrobial use decision-making process related to signs and symptoms specific, as well as specific diseases that they mention as important when deciding to treat with antimicrobials their animals
c. Previous experience	Farmers' previous experiences as reasons to decide the use of antimicrobials in their farms
d. Protocols and Standard Operating Procedures	Participant's comments about the role of protocols in the decision of treating an animal with antimicrobials in their farms
Subtheme 2: Modifiers	Despite that treating animals with antimicrobials is considered based on the previous reasons, some factors could potentially modify their decision of treating
a. Economics	Participants' comments about how economic factors influence their decision of treating an animal. Economic factors could be related with the value of the antimicrobial product, the value of the animal, milk production considerations, milk withdrawal, milk quota, etc
b. "Wait and see"	Participants' comments about how soon they usually treat an animal, or if there are specific circumstances when they prefer to wait a period of time to observe how the health of the animal evolve before treating with antibiotics
Subtheme 3: Information and referents for AMU	Participants' comments about the influence of other people or sources (i.e. media or meetings) in their decision of treating animals with antimicrobials

a. Veterinarian	Participants' mentions to their veterinarian in their health and management decisions including antimicrobials use. As well as their trust and confident feelings about their relationship with their veterinarian
b. Family and staff	Role of family and staff in the decisions related to health and management, including the antimicrobial use decisions
c. Other farmers	Participants' comments about the role of other farmers in their health and management decisions, including antimicrobial use
d. Other sources of information	Other people/sources not specified in the previous codes that have influence on their decision of treating animals with antimicrobials
**Theme 2: Reducing the use of antimicrobials on dairy farms**	This theme refers to the farmers' thoughts, attitudes, ideas, and barriers about the reduction of antimicrobial use in Canadian dairy industry
Subtheme 1: Attitudes toward reducing AMU on dairy farms	Participants expressed attitudes toward the idea of reducing antimicrobial use in the dairy industry
Subtheme 2: How to reduce AMU on dairy farms	Participants suggestions of how to reduce antimicrobial use in dairy industry
a. Better facilities and equipment	Participants suggestions of how to reduce antimicrobial use in dairy industry related to the improvement of facilities and equipment (i.e. new barn, improve housing, new milking system, etc.)
b. Herd management	Participants suggestions of how to reduce antimicrobial use in dairy industry related to the herd management (i.e. vaccination, genetic selection, facilities hygiene, etc)
c. New knowledge and information	Participants suggestions of how to reduce antimicrobial use in dairy industry related to the new information (i.e. additional training, veterinarian advices, meeting, conferences, etc)
Subtheme 3: Barriers	Real or perceived barriers expressed by participants to reduce the antimicrobials use in the dairy industry
**Theme 3. Antimicrobial resistance knowledge and perceptions**	This theme corresponds to all the participants' comments about antimicrobial resistance, experiences, perceptions, knowledge, and questions
Subtheme 1: Causes of antimicrobial resistance	Participants' knowledge, beliefs and questions about the causes of antimicrobial resistance

Subtheme 2. Impact of antimicrobial resistance	Participants' comments about the impact of antimicrobial resistance
a. Animal health and production	Participants' comments about the impact of antimicrobial resistance in animal and health production
Human health	Participants' comments about the impact of antimicrobial resistance in human health
b. Perception about AMR as a current problem	Participant perceptions about whether the AMR is a current problem or an overstated situation
